# Breaking the Mold: Surgical Exploration for Spinal Impalement Injury Without Neurological Deficit

**DOI:** 10.7759/cureus.39785

**Published:** 2023-05-31

**Authors:** Avinash Kachare, Jairam Jagiasi, Sudhir Sharan, Pravin U Jadhav, Kishor Munde

**Affiliations:** 1 Department of Orthopaedics, Lokmanya Tilak Municipal General Hospital and Medical College, Mumbai, IND

**Keywords:** broken knife, pseudomenigocoel, spine stab injury, spine trauma, impalment injury, spine

## Abstract

Spinal cord injuries (SCI) are a significant burden on society, particularly affecting the working population. Traumatic SCI can result from violent confrontations, involving firearms, knives, or edged weapons. Although surgical techniques for these injuries are not well defined, surgical exploration, decompression, and removal of the foreign body are currently indicated for patients with spinal stab wound injuries with neurologic impairment.

We present a case of a 32-year-old male patient who presented to the emergency department with a stab injury with a knife. Radiographs and CT scans revealed a broken knife blade with a midline trajectory in the lumbar spine, moving toward the vertebral body of L2 occupying less than 10% of the intramedullary canal. The patient underwent surgery, and the knife was successfully extracted without any complications. Post-operative MRI showed no signs of cerebrospinal fluid (CSF) leak, and the patient did not exhibit any sensorimotor deficit.

The acute trauma life support (ATLS) procedure must be followed while treating a patient with penetrating spinal trauma with or without neurological impairment. After availing appropriate investigations, any attempt to remove a foreign object should be done. Although spinal stab wound injuries are uncommon in developed nations, they continue to be a source of traumatic cord damage in underdeveloped countries. Our case highlights the successful surgical management of a spinal stab wound injury with a good outcome.

## Introduction

Spinal cord injuries (SCI) resulting in lifelong disabilities are a significant societal burden, particularly affecting the working population in their third decade of life [1]. The earliest known record of penetrating SCI dates back to 1700 BC in Egyptian papyrus, while in the second century [2], Greek physician Galen demonstrated that a horizontal cut through the spine of a monkey led to motor and sensory loss below the spinal cord cut [3]. Violent confrontations, typically involving firearms or knives, account for approximately 15% of traumatic SCI, with stab-wound injuries to the spine being rare in developed nations but still prevalent in underdeveloped countries [4]. Although surgical techniques for these lesions remain poorly defined due to their infrequency in affluent nations, surgical exploration, decompression, and foreign body removal are currently indicated for patients with spinal stab wound injuries and neurological impairment [5]. However, surgical exploration for individuals without neurological symptoms is controversial, given the risk of procedure-related injury.

Hereby, we present a case of successful surgical management of a spinal stab wound injury with knife extraction and a positive outcome in a 32-year-old male patient.

## Case presentation

**Figure 1:**
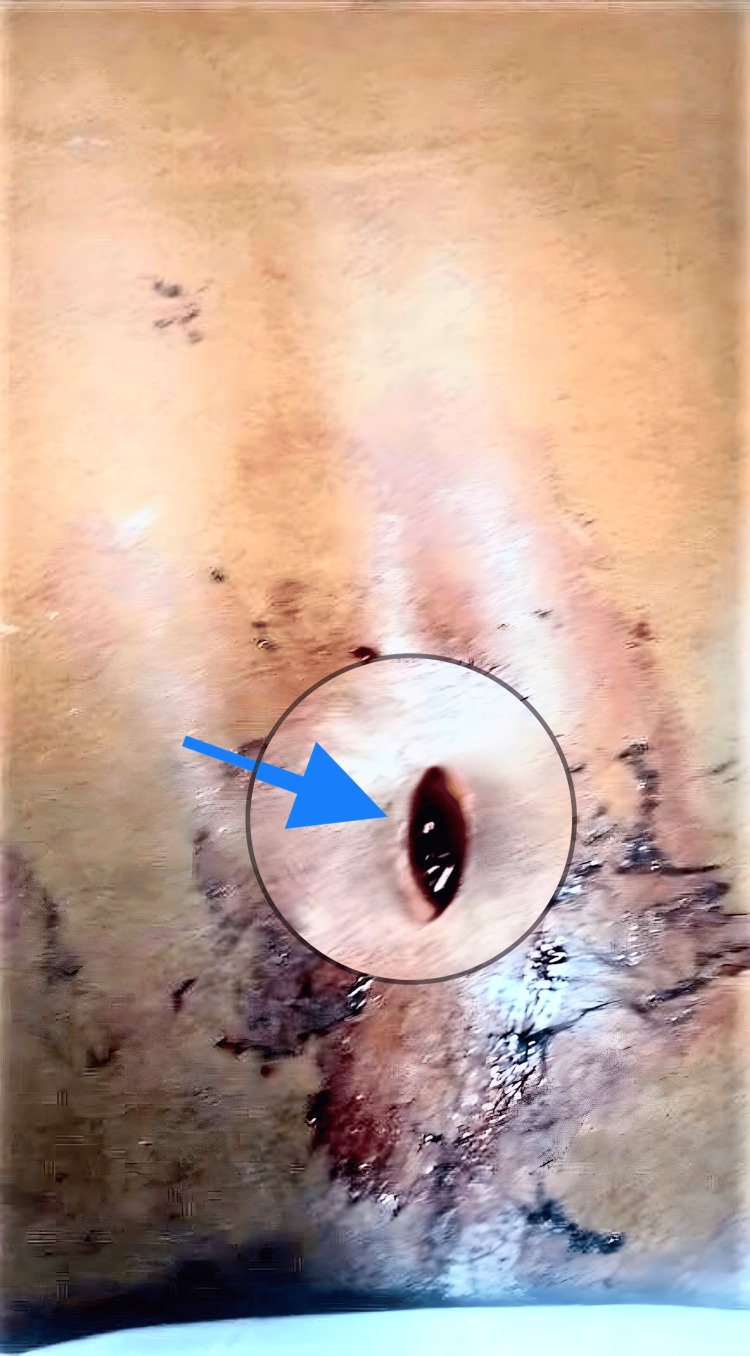
Elliptical stab wound marked by blue arrow and circle

A 32-year-old male patient with a history of stab injury with a knife presented to the emergency department four hours after the incident in a prone position on a trolley (Figure 1).

**Figure 2:**
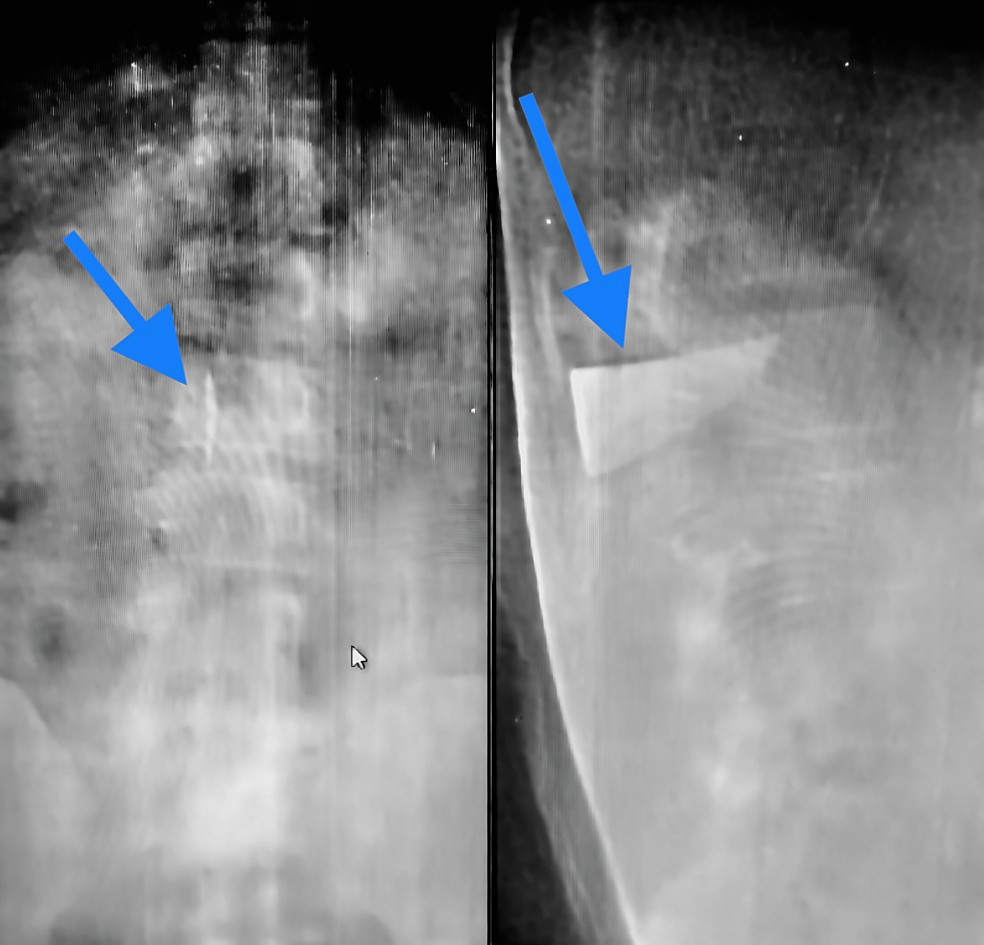
Foreign body on X-rays Antero-posterior and lateral X-ray view showing foreign body (knife blade) marked with a blue arrow

**Figure 3:**
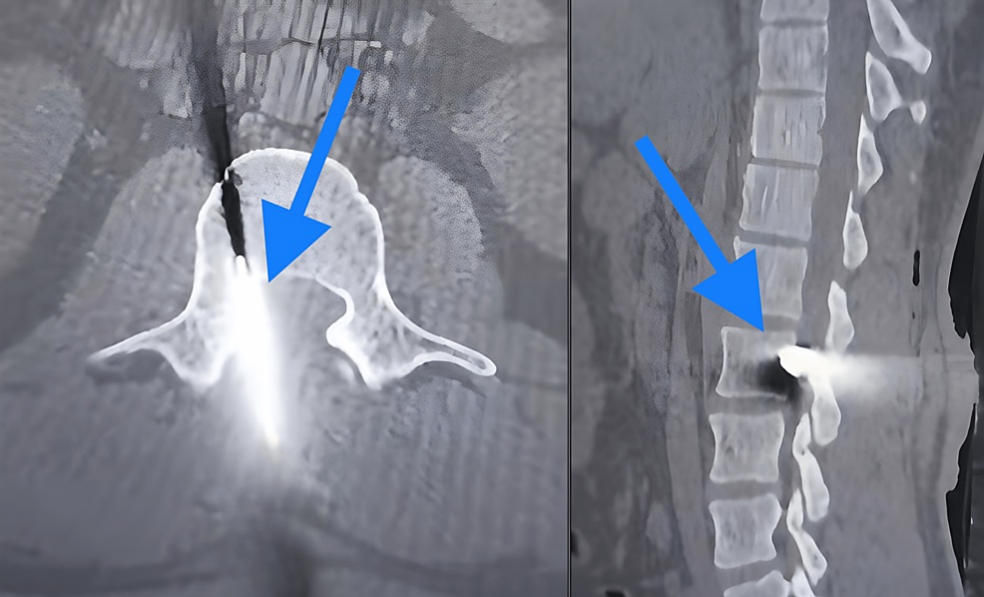
CT scan images Axial and sagittal CT cuts showing knife blade extending into the vertebral canal of the L2 vertebra

On admission, the patient was hemodynamically stable and had a Glasgow Coma Scale score of 15. Neurological evaluation revealed normal sensory function, full range of motion against resistance in the lower extremities, normal anal tone, and voluntary anal contraction. Radiographs (antero-posterior and lateral) and a CT scan were ordered to examine the position and route of the knife (Figure 2-3). The imaging studies showed a broken knife blade with a midline trajectory in the lumbar spine moving toward the vertebral body of L2, occupying less than 10% of the intramedullary canal. MRI was not performed due to the suspected ferromagnetic nature of the knife blade.

**Figure 4:**
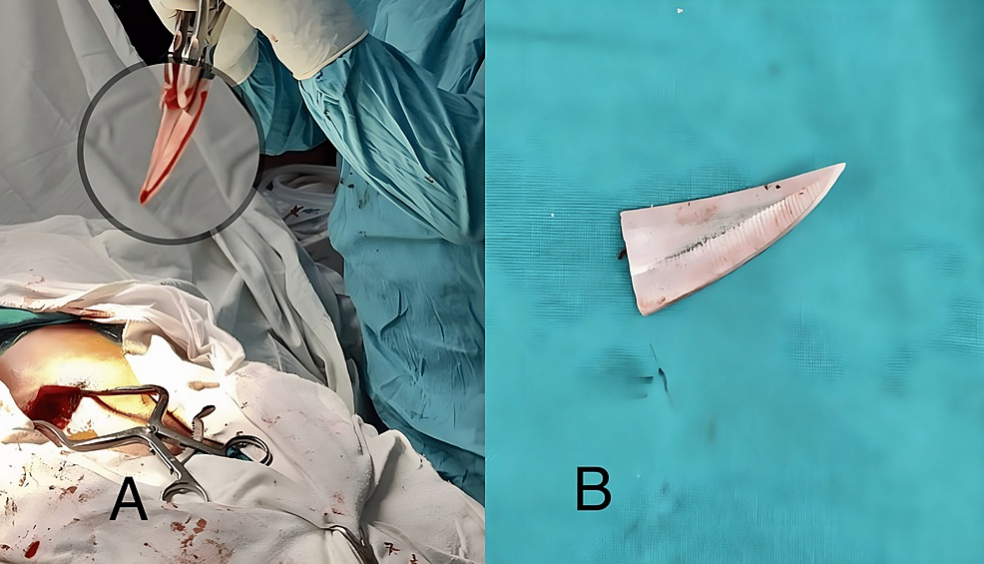
Surgical removal of Broken knife fragment A: removal of the knife blade B: knife blade (4.5x1.5 cm)

The patient was taken to the operating room and intubated under general anesthesia in the lateral position. The patient was then positioned in a prone position for subsequent surgery. The surgical exploration involved the removal of the L2 spinous process and a right-side L2 hemilaminectomy to expose the spinal canal. During the procedure, it was observed that the knife blade was passing through the lateral most part of the canal, while the dural sac remained intact without any nerve root injury. Great care was taken to protect the spinal cord and cauda equina nerve roots during the cautious removal of the broken knife. Notably, no dural tear or nerve root damage was detected. Subsequently, the wound was thoroughly irrigated with saline and debrided (Figure 4).

Under fluoroscopy guidance, the complete removal of the foreign body was confirmed, and there was no residual material in the spinal canal. The wound was closed using simple sutures. The patient received postoperative antibiotics but no steroids. Postoperatively, the patient had an uneventful recovery period, and there was no sensorimotor deficit. A postoperative MRI obtained showed no signs of cerebrospinal fluid (CSF) leak.

**Figure 5:**
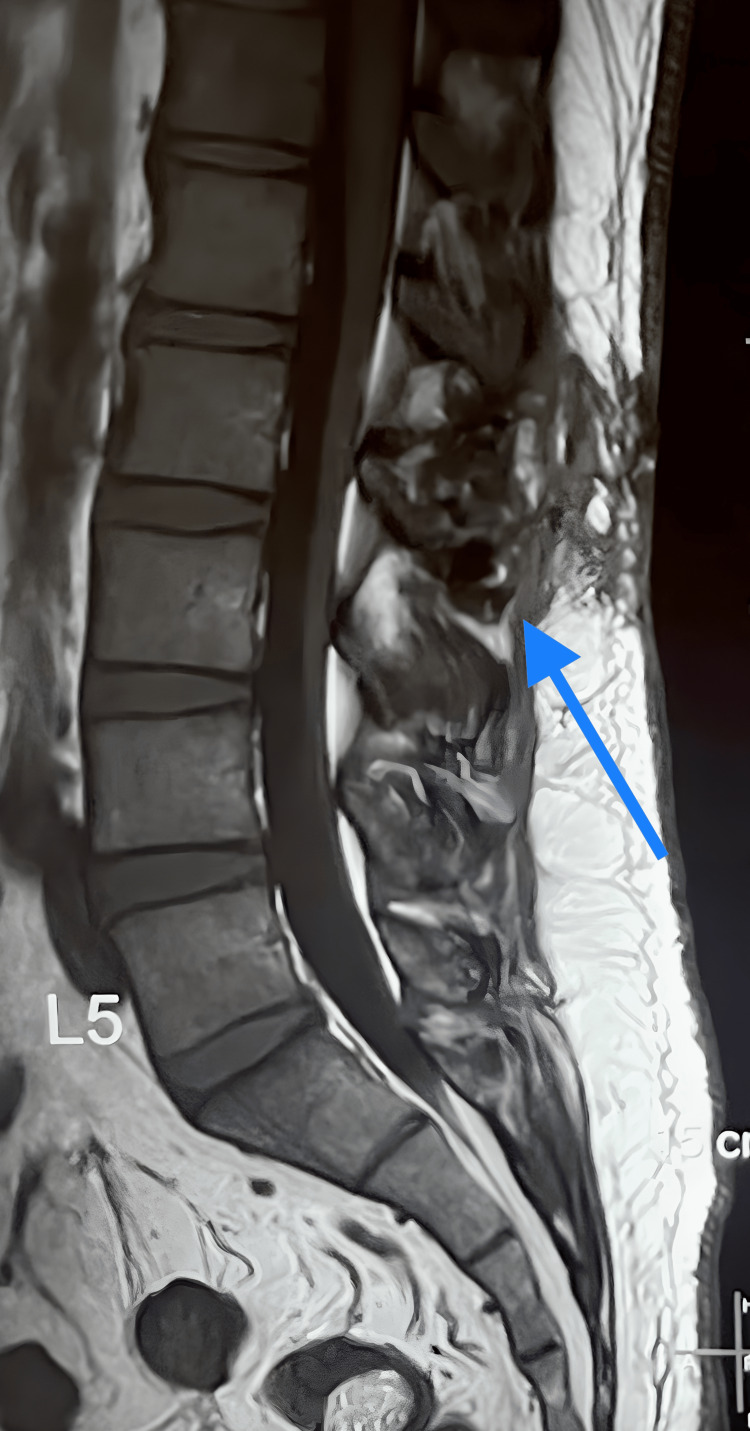
Follow-up T1 weighted MRI sagittal view Postoperative 18-month follow-up T1 weighted MRI image, arrowhead showing scarred soft region without any spinal cord structural abnormalities

**Figure 6:**
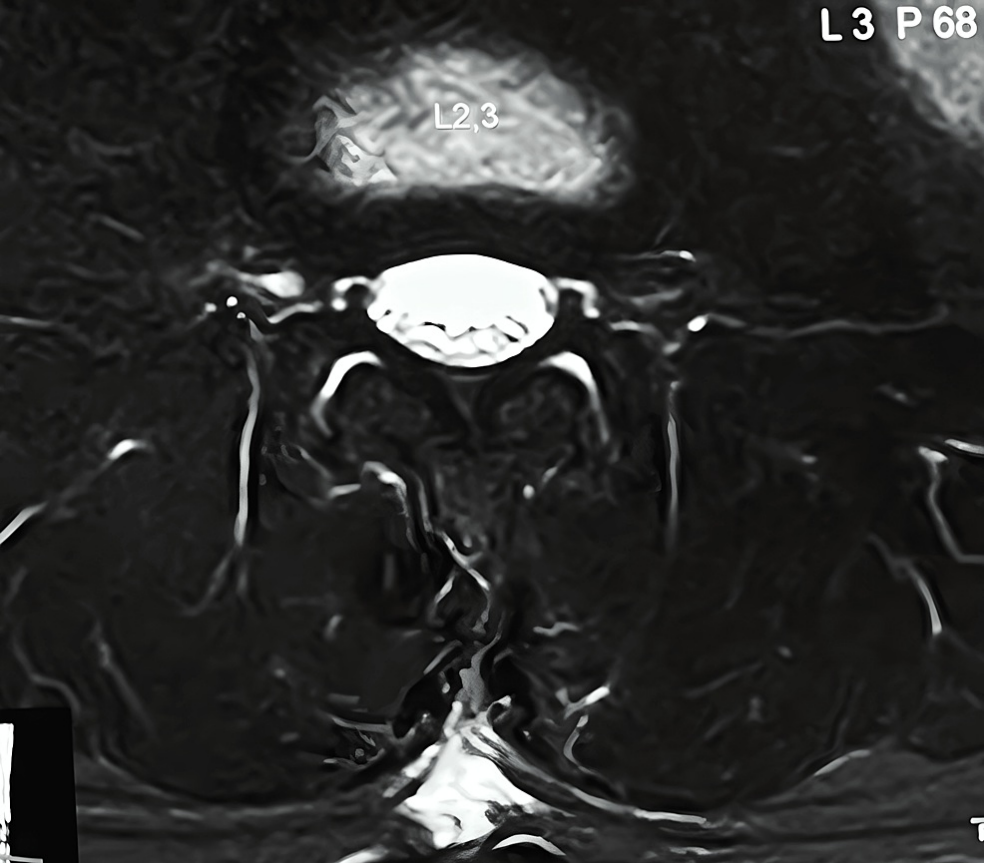
Follow-up MRI axial view Postoperative 18-month follow-up MRI axial view showing no structural changes in the spinal cord or nerve roots

The patient was followed up for 18 months, and an MRI was obtained to look for late sequelae. However, the MRI showed no complications (Figures 5-6).

## Discussion

The management of patients with penetrating spinal trauma, with or without neurological deficits, necessitates adherence to the advanced trauma life support (ATLS) protocol in order to preclude life-threatening injuries [6]. A thorough neurological assessment should be performed after patient stabilization. Removal of foreign objects should only be attempted once appropriate investigations have been conducted. The incidence of spinal impalement injuries is low, with limited research conducted on the topic; however, these injuries are associated with a high rate of neurological deficits [7]. Surgical intervention is mandatory if the patient presents with neurological deficits at admission or rapidly deteriorates. Late complications of penetrating spinal trauma have been documented, including myelopathy, intramedullary abscess, neurological impairment, spinal instability, pseudomeningocele, and granuloma [8].

The bulk of published data on SCI (60%) originates from South Africa during a period of heightened violence; however, in the last decade, there has been a shift in trends with an increasing number of cases being reported from North America [9]. Non-missile penetrating SCI can be caused by weapons such as knives, screwdrivers, scissors, and garden forks [10]. Unusual complications such as arachnoid and dural bleeds have been reported in minimally invasive acupuncture needle therapies [11]. Nevertheless, these were not observed in our patient prior to discharge and at the 18-month follow-up. Typically, the assailant stabs the victim and then withdraws the weapon, with the blade of the knife becoming trapped in the vertebral body or between the lamina, which may break in various places, causing the knife to present as a foreign body. A similar mechanism was observed in our case, where the handle of the knife broke due to the victim's sudden turning during the assault, and the knife blade was retained in the vertebra and spinal canal. NMPSCI exhibits the following mechanisms of injury: immediate direct damage to nervous tissue, severing of vertebral arteries, artery of Adamkiewicz resulting in complete SCI or infarction, spinal hematoma, or foreign objects causing pressure effects on the spinal cord. In the delayed phase, there may be CSF leak, cord edema, granuloma, scars, and pseudomeningocele [12].In NMPSCI, investigations should be conducted to determine the spatial position of foreign bodies and to rule out other injuries. The importance of planar radiographs and CT scans cannot be overstated. Because of the knife's ferromagnetic characteristics, MRI should be postponed. Angiography should be used to rule out any possibility of bigger vessel damage [13]. Antibiotics are the gold standard for preventing infections; however, steroid usage is not recommended in NMPSCI [14]. The mode of transportation for a patient with an impaled knife in situ presents a challenge. The patient should be kept in the lateral or prone position, and intubation can be performed in the lateral position. The patient should be moved as a single unit, minimizing intersegmental motion. The primary response team should not be distracted by the impaled knife and should not skip the ATLS protocol [6].

It is our opinion that the removal of a knife should be carried out in a controlled environment, such as an operating room. Even if radiographic imaging indicates no foreign bodies present after direct withdrawal, surgical exploration and wound debridement should be performed to search for and extract any non-radiolucent foreign bodies, such as wood, plastic, or cloth fragments, which may remain [15]. Surgical intervention has been demonstrated to reduce delayed complications. Delayed wound infection has been observed in cases managed solely with irrigation and suture [1]. Decompression, laminectomy, and hemilaminectomy are typically required for symptomatic cases. In cases of rapidly progressing neurological deficiencies and incomplete SCI, extensive exploration is advised [16]. For optimal symptom relief, sufficient canal decompression is necessary. When approaching the spinal cord, the uninjured region should be addressed before the injured region and then moved further toward the uninjured region [9]. CSF leak, infection (less than 1% progress to chronic abscess and osteomyelitis), and rarely meningitis; chronic epidural granulation (which may present as progressive myelopathy); and arachnoiditis, pseudomeningocele, and syringomyelia are all reported complications related to the spine [8]. Metal particles such as copper or silver can create a severe inflammatory response, while nickel and lead particles may elicit a more moderate reaction. Pseudomeningocele can arise due to the severing of nerve sleeves and continuous unidirectional CSF flow, and as a late complication, it may result in neurological deficiency [9].

Penetrating SCI patients require a lengthier hospital stay and greater hospital expenditures than nonpenetrating SCI patients [17]. A recent systematic review concluded that surgical and conservative management for PSCI has equivalent effectiveness regardless of injury severity, emphasizing the need for tailored treatment strategies and careful surgical selection [18]. Zhang et al. advised the management of penetrating spinal cord injury with retained foreign bodies by case-to-case assessment, highlighting the importance of immediate surgical removal to prevent contamination [19]. A recent literature review and meta-analysis found no clear benefit to surgical decompression for penetrating SCI caused by shrapnel, emphasizing the need for further nonrandomized prospective cohort studies on decompression and stabilization surgery in blast injuries [20].

Our case highlights the importance of prompt surgical intervention and removal of foreign objects in patients with spinal stab wound injuries, demonstrating successful management and positive outcomes in a potentially debilitating condition such as spinal stab injury.

## Conclusions

To minimize early or late complications, we suggest that neurological implement damage should be surgically explored, while neurological deficiency warrants a more extensive surgical approach such as decompression. Over the course of the 18-month follow-up, our patient experienced no complications, including pseudomeningocele, meningitis, or any late wound complications.
